# Single-Cell RNA Sequencing Reveals Smooth Muscle Cells Heterogeneity in Experimental Aortic Dissection

**DOI:** 10.3389/fgene.2022.836593

**Published:** 2022-08-11

**Authors:** Cheng Xu, Xiaowei Liu, Xiaoxin Fang, Lei Yu, Hui Chong Lau, Danlei Li, Xiaoman Liu, Haili Li, Justin Ren, Baohui Xu, Jianjun Jiang, Lijiang Tang, Xiaofeng Chen

**Affiliations:** ^1^ Department of Cardiology, Taizhou Hospital Affiliated to Wenzhou Medical University, Taizhou, China; ^2^ Department of Cardiology, Zhejiang Hospital, Hangzhou, China; ^3^ Department of Medicine, Crozer-Chester Medical Center, Upland, PA, United States; ^4^ Department of Respiratory and Critical Care Medicine, The First Affiliated Hospital of Guangxi Medical University, Nanning, China; ^5^ Department of Surgery, Stanford University School of Medicine, Stanford, CA, United States; ^6^ Department of Radiation Oncology, Indiana University School of Medicine, Indianapolis, IN, United States

**Keywords:** aortic dissection, single-cell RNA sequencing, gene ontology enrichment analysis, smooth muscle cells, KEGG enrichment analysis

## Abstract

**Purpose:** This study aims to illustrate the cellular landscape in the aorta of experimental aortic dissection (AD) and elaborate on the **smooth muscle cells (**SMCs**) heterogeneity** and functions among various cell types.

**Methods:** Male Apolipoprotein deficient (ApoE^−/−^) mice at 28 weeks of age were infused with Ang II (2,500 ng/kg/min) to induce AD. Aortas from euthanized mice were harvested after 7 days for 10×Genomics single-cell RNA sequencing (scRNA-seq), followed by the identification of cell types and differentially expressed genes (DEGs). Gene Ontology (GO) enrichment and Kyoto Encyclopedia of Genes and Genomes (KEGG) analysis was conducted.

**Results:** AD was successfully induced in ApoE^−/−^ mice. scRNA-seq identified 15 cell clusters and nine cell types, including non-immune cells (endothelials, fibroblasts, and SMCs) and immune cells (B cells, natural killer T cell, macrophages, dendritic cells, neutrophils, and mast cells). The relative numbers of SMCs were remarkably changed, and seven core DEGs (ACTA2,IL6,CTGF,BGN,ITGA8,THBS1, and CDH5) were identified in SMCs. Moreover, we found SMCs can differentiate into 8 different subtypes through single-cell trajectory analysis.

**Conclusion:** scRNA-seq technology can successfully identify unique cell composition in experimental AD. To our knowledge, this is the first study that provided the complete cellular landscape in AD tissues from mice, seven core DEGs and eight subtypes of SMCs were identified, and the SMCs have evolution from matrix type to inflammatory type.

## Introduction

Aortic dissection (AD) is a life-threatening aortic dilatation disease with a significant fatality rate as high as 1–2% per hour after symptom onset in untreated patients (Gawinecka et al., 2017). Epidemiological data revealed that 93.5% of AD patients in China are under the age of 70, with an average age of onset of 52 years-old (Wang et al., 2014). The pathophysiology behind remains unclear and the main clinical treatment modalities are surgery and endovascular repair as effective drug treatment are still unavailable to stop the progression (Wang et al., 2018).It necessary to have a deeper understanding of the pathogenesis of AD to improve clinical outcomes. The occurrence and development of AD are complex, comprising of gene mutation, hypertension, blood lipid level, angiotensin II (Ang II) and inflammation (Fan et al., 2014; Qi et al., 2018; Renard et al., 2018; Tanaka et al., 2018; Wolford et al., 2019). Common pathological changes of AD patients include extensive destruction of vascular elastic fiber dimension, loss of the adventitia elastic fiber and neutrophil infiltration (Yuan and Wu, 2018).However, the cell types contributing to AD progression have not been fully elucidated. Single-cell RNA sequencing (scRNA-seq) technology is to sequence the RNA of a single cell, which can generate the unique transcription of an individual cell at single-cell resolution and establish a gene regulation network (Lambrechts et al., 2018; Han et al., 2020). The cellular heterogeneity of aneurysmal abdominal aorta has been described by scRNA-seq (Zhao et al., 2021). A mouse endothelial cell map and the heterogeneity of mouse endothelial cells has also been reported recently (Kalucka et al., 2020). In our current study, we successfully constructed a mouse model of AD by using Ang II followed by scRNA-seq technology to clarify the diversity of the cell profiles of vascular tissue and identify marker genes for different cell clusters. We further analyzed the smooth muscle cells (SMCs) and clarified the core genes of AD formation and development trajectory, which provided a new perspective on the complex biological systems and key functions associated with disease development.

## Methods

### Mouse Model

28-weeks-old male ApoE^−/−^ mice were purchased from Zhejiang Academy of Medical Sciences and raised in the animal room of Zhejiang Academy of Medical Sciences under specific pathogen-free (SPF) environment. Mice were housed in a temperature-controlled room at 25 ± 2°C and humidity of 55 ± 3%, under 12 h light and dark cycle. ApoE-^/-^ mice were randomly divided into two groups, with 15 mice in each control and study group, infused for 7 days with Ang II (2500 ng/kg/min) and saline respectively.

### Ultrasonography Examination

After 7 days of aorta modeling, ultrasound was used to evaluate the formation of abdominal AD. A MS550 S probe on the small animal ultrasonic imaging system was adjusted at center frequency of 40MHz, focal length of 6 mm, fixed focus depth of 10 mm, and width of 12.00 mm. After the mice were anesthetized with 1.5–2% isoflurane gas, the hair from the chest to the abdomen of the mice was removed with depilation cream followed by application of a warm paper towel to wipe dry the depilated skin. After fixating the mice on the examination table, ultrasonic gel was applied to the area of interest prior to the ultrasonography examination.

### Specimen Collection and Pathological Examination

After the ultrasonography examination was performed, the aorta tissues were removed and preserved in 4% paraformaldehyde solution for 24 h. The tissues were then embedded in paraffin solution and subsequently sectioned evenly to the pieces of 5 μm thickness. The specimens were then stained with hematoxylin-eosin (H&E) and van Gieson (EVG)stains and examined using a light microscope.

### Aortic Vascular Tissue Procurement and Isolation

The harvested aortic tissue was washed three times with 0.9% normal saline on ice**.** The samples were washed with a medium and appropriate amount of enzyme (1X, Gibco, 12605010) were added followed by incubation for 1 h. After enzyme digestion, the samples were placed on a stainless steel cell filter and washed with culture medium for two times followed by centrifugation at 4°C and 300 g for 5 min. Red blood cells were subsequently removed using 1X RBC lysis buffer (Cat: R1010, Solarbio, China) for 5 min and cells were centrifuged at 4°C and 300 g for 5 min. The final samples were washed with medium for two times.

### Single-Cell RNA Sequencing

Sc-RNA seq was performed using the 10x Genomics’ single-cell solution. Single-cell suspensions from 4 aortas in control group were pooled together as one sample and three aortas in experimental group were pooled together as another sample respectively. The emulsion and the library were prepared according to the user guide of 10x Genomics Chromium Single Cell 3’v3 Reagent Kit. GEMs (Gel Bead-in-Emulsions) are generated by combining specimens, a Master Mix, gel beads and partitioning oil, onto the chip in the chromium single cell controller kit. After the cell is lysed and RNA reverse transcription occurs, the cDNA was purified from the mixture. The cDNA was amplified, fragmented, and spliced to generate sufficient mass for library construction. The cDNA was subsequently sequenced by Illumina sequencer.

### Single-Cell RNA Sequencing Data Analysis

A criterion to filter out cells with UMI/gene numbers out of the limit of mean value +/- 2 fold of standard deviations was applied to remove cells of low quality and likely multiple captures, which is a major concern in microdroplet-based experiments. Low-quality cells where >10% of the counts belonged to mitochondrial genes were further discarded following visual inspection of the distribution of cells by the fraction of mitochondrial genes expressed.

### Identification of Cluster Marker Genes and Cell Types

Principal component analysis (PCA) was performed to reduce the dimensionality with RunPCA function in Seurat (Butler et al., 2018).A 2-dimensional t-distributed stochastic neighbor embedding (t-SNE) algorithm with the RunTSNE function in Seurat was used to identify cell clusters (Butler et al., 2018). The FindAllMarkers function (test.use = bimod) in Seurat helps to identify marker genes of each cluster. We used the R package SingleR (Aran et al., 2019), a novel computational method for unbiased cell type recognition of scRNA-seq with Mapping the Mouse Cell Atlas by Microwell-Seq as a reference to infer the cell origin of each of the single cells independently and identify cell types (Han et al., 2018).

### Identification of DEGs

The FindMarkers function (test.use = MAST) in Seurat (Butler et al., 2018) was utilized to identify differentially expressed genes (DEGs) between aortic dissection group and normal control group samples in RStudio.The criteria were as follows: (1) the *p* < 0:01; (2) log2FoldChange≥1.5 or ≤ -1.5.

### Protein-Protein Interaction Analysis

In order to determine the core genes in the DEGs of SMCs, we used online tool STRING (version 11.5) (http://string-db.org/) to construct the PPI network, and the parameters were set to the default value.

### Hub Genes Selection and Analyses in SMCs

The cytohubba (Chin et al., 2014) plug-in in Cytoscape was used to screen the core genes in PPI network, We randomly selected 5 of the 12 algorithms in the cytohubba plugin, and then used the intersection of the results of the 4 algorithms to determine the hub gene. After screening the core gene, we performed Gene Ontology (GO) and Kyoto Encyclopedia of Genes and Genomes (KEGG) enrichment analysis to determine the function of the core gene.

### GO Enrichment Analysis and KEGG Pathway Analysis

GO and KEGG pathway enrichment analyses for DEGs in SMCs was performed using the R package clusterProfile (Yu et al., 2012), which provided a comprehensive set of functional annotation tools allowing investigators to understand the biological meaning behind a large list of genes.

### Reconstructing SMCs Differentiation Trajectories by Monocle2

SMCs pseudotime trajectories were reconstructed by the Monocle2 R package (version 4.1.2). Briefly, the work flow chart is as follows: (1) the types of SMCs were selected by Seurat; (2) Reduces the high-dimensional expression profiles to a low-dimensional space; (3) Single cells are projected onto this space and ordered into a trajectory with branch points.

## Results

### Ultrasonographic Results

**FIGURE 1:**
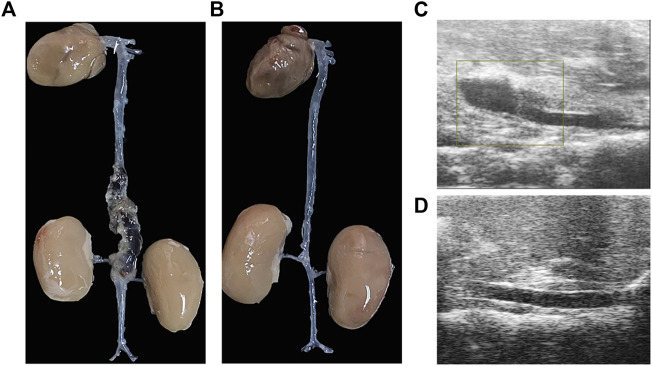
The aortic dissection induced by angiotensin II infusion. **(A)** Gross morphology of aortic dissection. **(B)** Gross morphology of normal blood vessels. **(C)**Ultrasound image of aortic dissection in the experimental group. **(D)** Ultrasound image of normal vessels in the control group.

After Ang II infusion, there was dilatation of the aorta, progressing to separation of the blood vessel walls, and subsequently forming a false lumen. This was consistent with the ultrasound findings of aortic dissection (Figures 1A,C), while the vascular wall of the abdominal aorta was normal in the control group (Figures 1B,D).

### Pathological Examination

**FIGURE 2:**
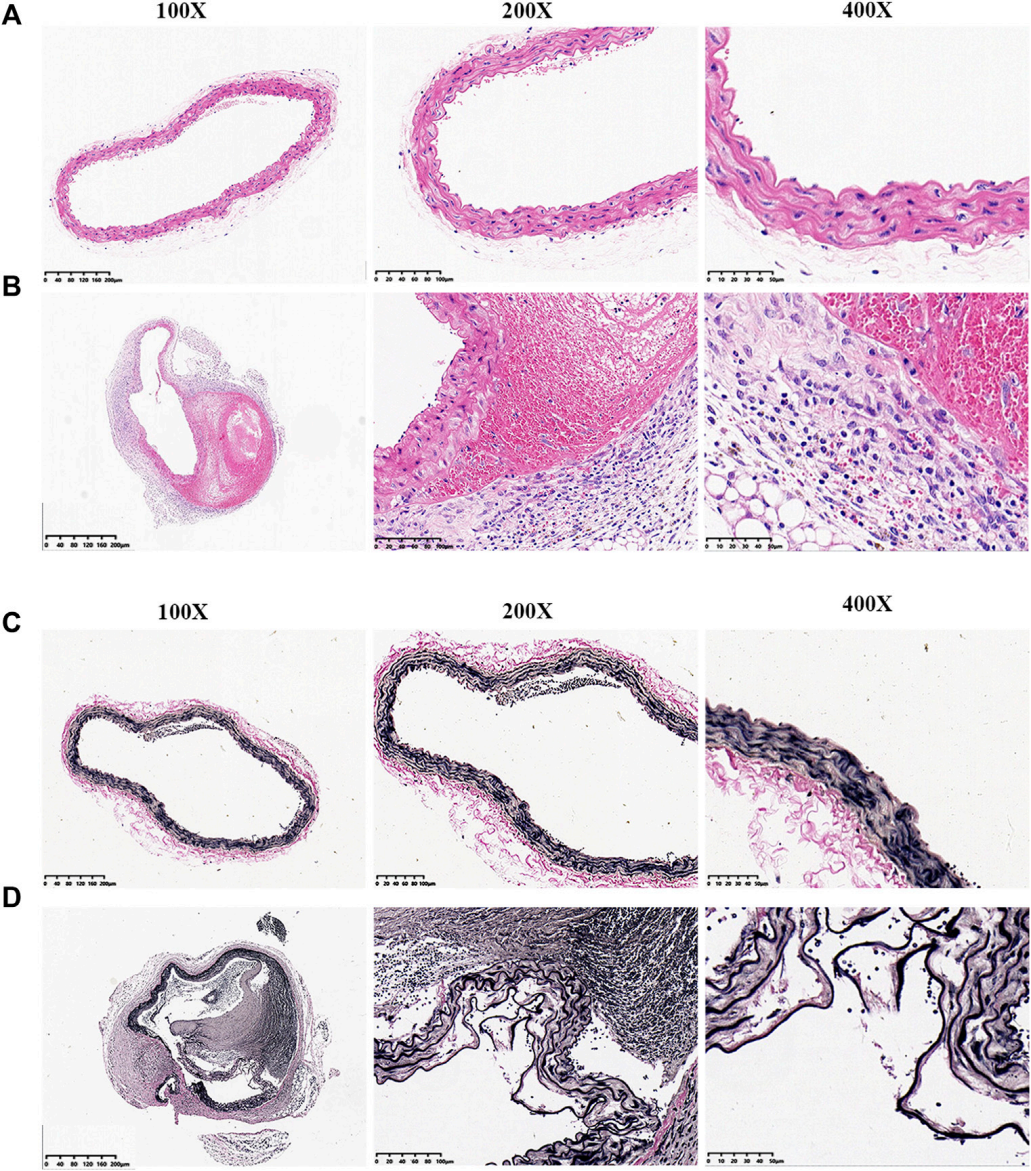
EVG and H&E staining of aortic vessels in mice from each experimental group. **(A)** H&E staining of normal blood vessels. **(B)** H&E staining of aortic dissection blood vessels. **(C)** EVG staining of normal blood vessels. **(D)** EVG staining of aortic dissection blood vessels.

Aortic sections were stained with H&E and EVG. In the control group, vascular wall structure had no changes with a well-organized smooth muscle layer and no change in wall thickness or formation of pseudo-lumen (Figures 2A,C). In the study group, the vascular intima was torn with a decreased wall thickness. Medial elastic fibers were disordered in conjunction with adventitial inflammatory cell infiltration, intramural thrombus and pseudo-lumen (Figures 2B,D).

### Single-Cell RNA Sequencing Clustering Analysis

**FIGURE 3:**
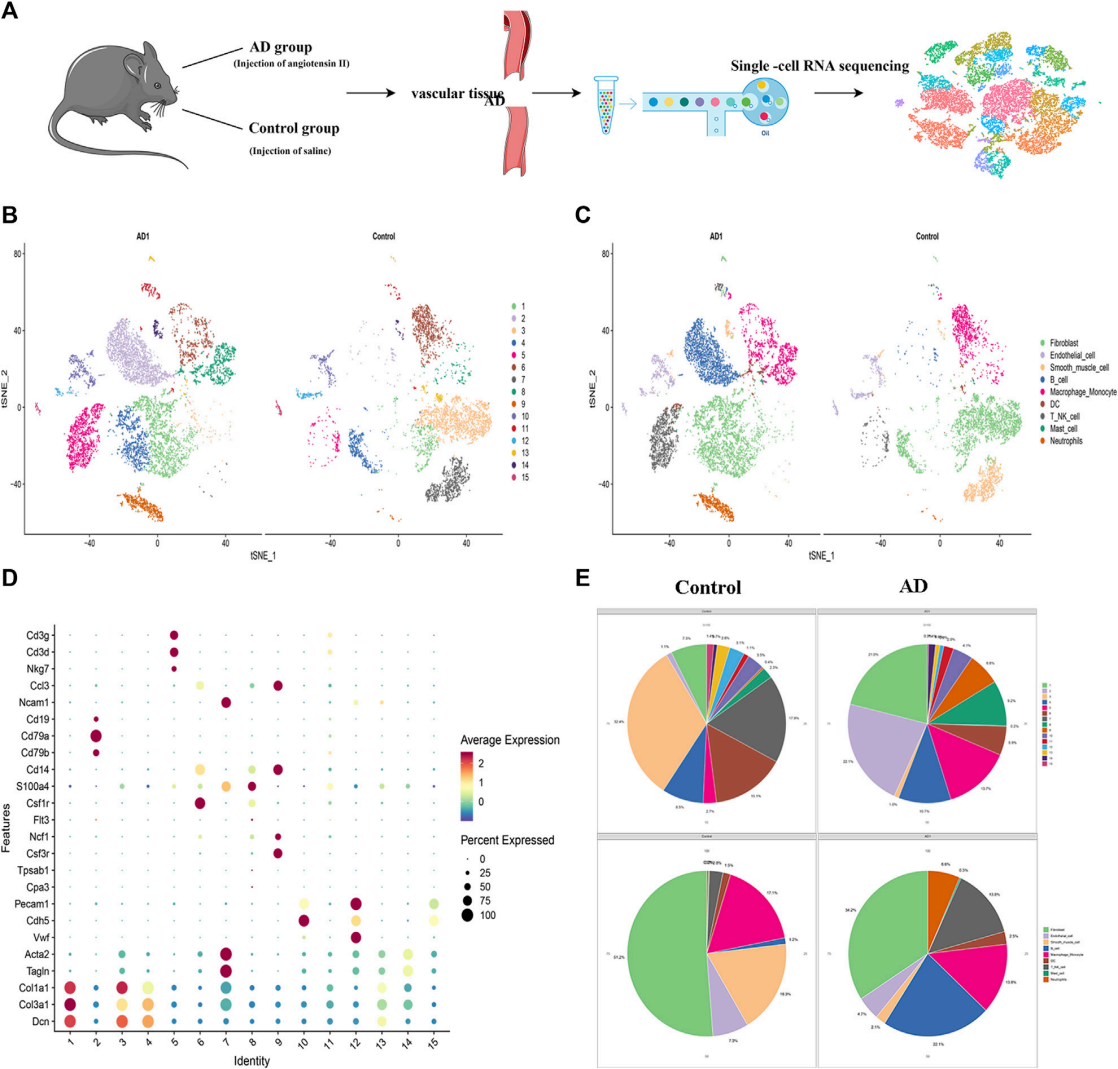
Identification of cell clusters and types by single-cell RNA sequencing (scRNA-seq). **(A)** Schematic of the experimental design for single-cell RNA sequencing. **(B)** Distribution of cells in AD and control groups.**(C)** t-SNE plot demonstrated nine cell types. **(D)** the key marker genes in 15 clusters.**(E)** Pie chart of cell types and clusters.

SCRNA-seq analysis was performed on vascular tissue from 2 groups as illustrated in Figure 3A. 18,835 cells were used for integrated single-cell RNA-Seq analysis. Based on the similarity of gene expression, 15 clusters of cells and nine cell types were identified (Figures 3B,C). The key marker gene of each cell type was shown in Table 1. The major cell types comprised of: (i) Fibroblasts (Clusters1,2); (ii) Endothelial cells (Clusters 9,13,14); (iii) Smooth muscle cells (Cluster 6,12); (iv) B cells (Clusters 3); (v) Macrophage & Monocyte (Cluster 4,8); (vi) DC (Cluster 11); (vii) NK-T cells (Cluster 5,15) (viii) Mast cells (Cluster 8); (ix) Neutrophils (Cluster 7), the key marker genes for each cell cluster are presented in Figure 3D.

**TABLE 1:** Marker genes in different cell types.

Cell type	Cluster	Marker genes
Fibroblast	1, 2	COL1A1, COL3A1, DCN
Endothelial	9, 13, 14	PECAM1, CDH5, VWF
Smooth muscle cells	6, 12	ACTA2,TAGLN
B cell	3	CD19, CD79A, CD79B
Macrophage & Monocyte	4, 10	CD14,S100A4,CSF1R
DC	11	FLT3
NK-T	5, 15	CCL3,NCAM1
Mast cell	8	TPSAB1,CPA3
Neutrophils	7	NCF1, CSF3R

### Differences in Cell Type Among Control and AD Groups

The 6,323 cells from the control group were assigned to nine different cell types (Figure 3E) with the following proportions: 1.20% of B cells, 1.5% of DC, 7.3% of endothelial cells, 51.2% of fibroblast, 17.1% of macrophages and monocyte, 0.16% of mast cells, 0.35% of neutrophils, 2.78% of NK-T cell, and 18.31% of SMCs. In AD group, 10756 cells were clustered depending on the similarity of gene expression, and 9 cell types were identified. The main cell types with their corresponding proportions were as follows: 22.1% of B cells, 2.5% of DC, 4.7% of endothelial cells, 34.2% of fibroblasts, 13.8% of macrophages and monocytes, 0.30% of mast cells, 6.56% of neutrophils, 13.85% of NK-T cells, and 2.06% of SMCs. Besides, we noticed significant changes in SMCs, which decreased from 18.3% of normal tissue to 2.1% of dissection tissue, In our previous study, the expression of Extracellular matrix metalloproteinase inducer (EMMPRIN) in SMCs will significantly affect the progress of AD (Chen et al., 2009), this suggests that SMCs may play an important role in AD and deserves priority attention.

### Identification of DEGs and PPI Network Construction in SMCs

**FIGURE 4:**
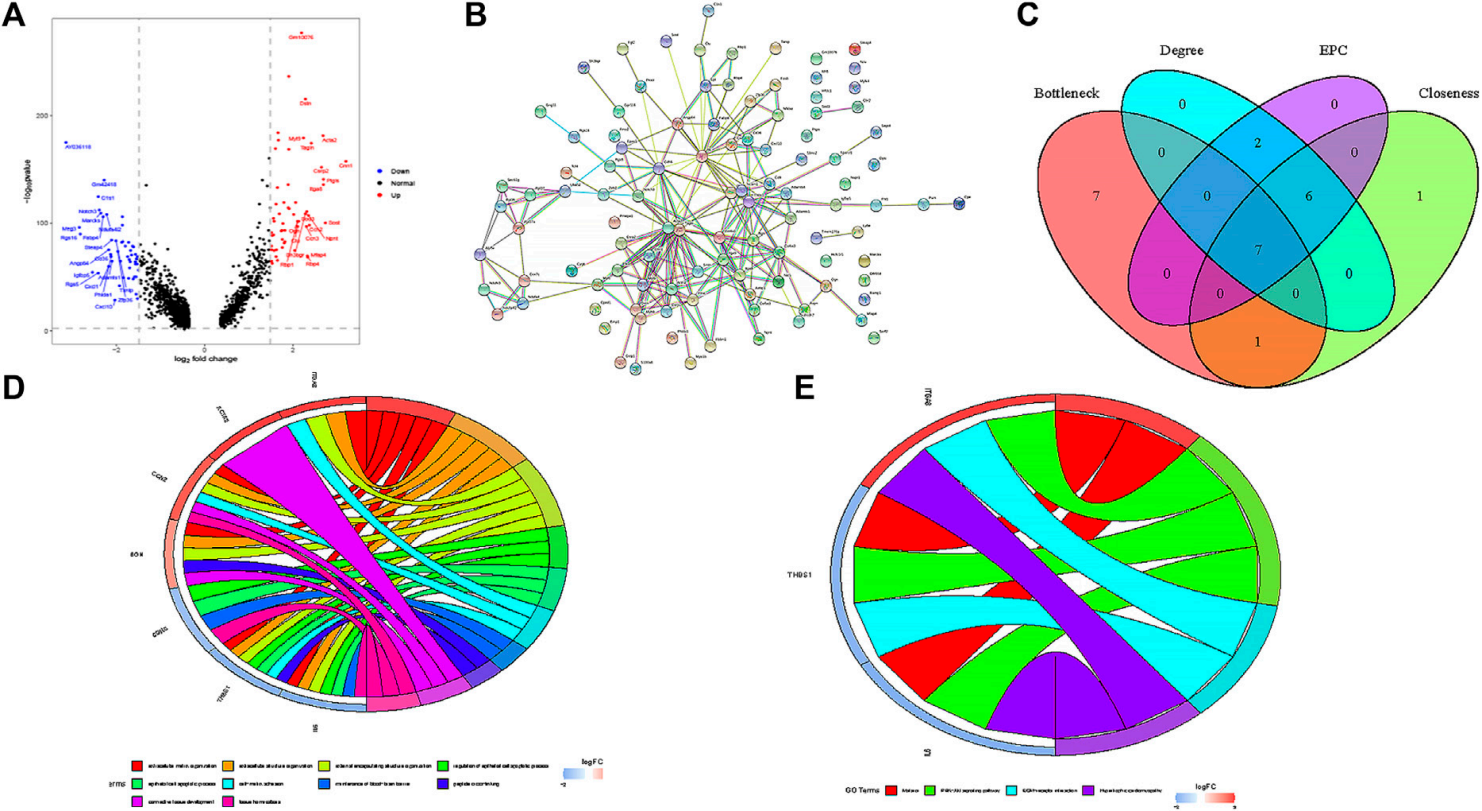
Functional analysis of smooth muscle cells. **(A)** Volcanic map of DEGs in smooth muscle cells. **(B)** PPI network of DEGs. **(C)** seven hub genes were identified by overlapping the first 15 genes in the four classification methods of cytoHubba. **(D)** GO enrichment analysis of seven hub genes. **(E)** KEGG enrichment analysis of seven hub genes.

In SMCs, we identified 59 up-regulated genes and 51 down-regulated genes (Figure 4A), After importing DEGs into STRING database, an interaction network with 86 nodes and 440 sides was obtained (Figure 4B).

### Hub Gene Selection and Analysis

In this study, we used cytohubba to identify core genes, according to the four algorithms in cytohubba (Bottleneck, Degree, EPC, Closeness), the ranking of the top 15 core genes selected by each algorithm is shown in Table 2. Finally, seven central genes (ACTA2, IL6, CTGF, BGN,ITGA8,THBS1, and CDH5) were identified by overlapping the15 genes (Figure 4C). GO and KEGG enrichment analyses were conducted based on the seven central genes of SMCs. The significant GO terms of core genes were “extracellular matrix organization”, “extracellular structure organization”, and “regulation of epithelial cell apoptotic process” (Figure 4D), and indicates that these seven core genes play an significant role in changing the function of extracellular matrix. In KEGG enrichment analysis, only three core genes (ITGA8, THBS1, and IL6) were enriched in four signal pathways: “malaria”, “PI3K−Akt signaling pathway”, “ECM−receiver interaction”, and “hypertrophic cardiopathway” (Figure 4E), which imply that these genes play a role in promoting metabolism, growth and angiogenesis in SMCs.

**TABLE 2:** The Top 15 Hub Genes Rank in cytoHubba.

Bottleneck	Degree	EPC	Closeness
**ACTA2**	**ACTA2**	**ACTA2**	**ACTA2**
**IL6**	**IL6**	TAGLN	**IL6**
**CTGF**	TAGLN	**IL6**	**CDH5**
MYL6	**CDH5**	**CDH5**	**THBS1**
NOTCH3	**THBS1**	**THBS1**	TAGLN
**BGN**	**BGN**	**BGN**	**BGN**
UBA52	CLO4A1	ACTN1	**CTGF**
**ITGA8**	ACTN1	COL4A1	COL4A1
**THBS1**	**CTGF**	**CTGF**	NOTCH3
**CDH5**	ITGA9	FLNA	**ITGA8**
IGFBP5	FLNA	**ITGA8**	ITGA9
NDUFA4	**ITGA8**	ITGA9	ACTN1
RGS5	COL6A3	MYL9	CXCL1
FHL1	MYL9	CNN1	FLNA
NID1	CNN1	COL6A3	MYL9

Bold characters represent the common genes screened by the four algorithms.

### The Trajectory Analysis of SMCs in AD

**FIGURE 5:**
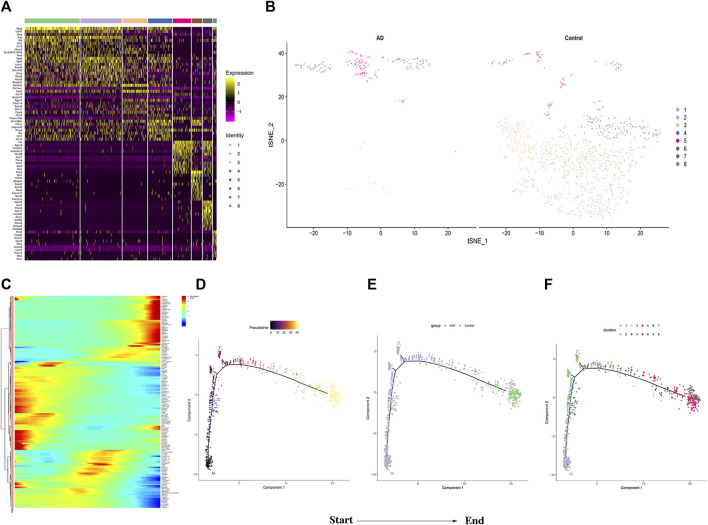
Analysis of smooth muscle cell subgroup .**(A)**Heatmap of the top 10 enriched genes in each SMCs population. **(B)** Distribution of SMCs Subgroup between the two groups. **(C)**The heatmap of top 50 genes which had most critical influences on cell transformation. **(D)** Time progression of SMCs differentiation. **(E)** and **(F)** SMCs differentiation trajectory.

**FIGURE 6:**
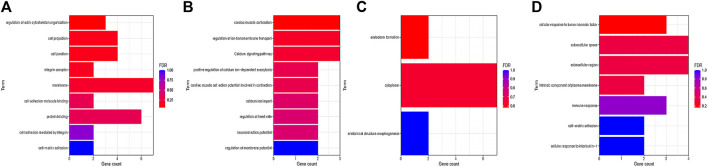
GOanalysis of smooth muscle cell subtypes. **(A)** GO enrichment analysis of SMC2. **(B)** GO enrichment analysis of SMC6. **(C)** GO enrichment analysis of SMC7. **(D)** GO enrichment analysis of SMC5.

The changes of cell phenotype and function occur gradually. Due to the great changes in the number of SMCs, we applied Monocle2 to perform pseudotime trajectories of all SMCs and showed the differentiation direction between them. Through the expression of characteristic genes, all SMCs could be divided into 8 types (Figures 5A,B), and the marker genes of each subtype are shown in Supplementary Material. SMCs mainly differentiate from SMC2 to SMC5 and undergo a variety of phenotypic changes (Figures 5E,F). Besides, we divided 8 kinds of SMCs into three modules according to the changes of gene expression. Module1:The expression of genes first increased and then decreased with the development of AD; Module2: The expression of genes decreased with the development of AD; Module3: The expression of genes increased with the development of AD (Figure 5C). In addition, it can be seen from Figure 5F that SMCs differentiate from SMC2 and are finally divided into SMC5, SMC6 and SMC7. In order to understand the role of each SMC subtype, we further performed GO function analysis.GO enrichment analysis showed that SMC2 was mainly related to “regulation of actin cytoskeleton organization”, “cell junction”, and “cell adhesion molecular binding”, (Figure 6A), suggesting that it was a stromal SMC; SMC6 is mainly associated with “regulation of calcium ion transmembrane transport”, “calcium signaling pathway”, and “positive regulation of calcium ion-dependent exocytosis” (Figure 6B), suggesting that it is a signal transduction type smooth muscle cell; SMC7 is mainly related to “endoderm formation”, “cytoplastmm” and “analytical structure morphogenesis” (Figure 6C). We believe that SMC7 is a transitional SMC after SMC6 signal transduction. SMC5 is related to “cellular response to tumor necrosis factor”, “immune response”, “cellular response to interleukin −1″, suggesting that it is an inflammatory SMC (Figure 6D).

## Discussion

AD is a medical emergency. However, the underlying mechanism of the disease has remained unclear. Currently, there is no identifiable effective pharmacologic management to treat AD. In this study, we successfully constructed an AD model using Ang II with an input of 2,500 ng/kgmin within 7 days. The histopathological results of the model constructed demonstrated similar pathological manifestations of AD in humans, including tearing of tunica intima, rupturing of elastic intima, and infiltration of inflammatory cells on the adventitia (Del Porto et al., 2010; Wang et al., 2020).

The present study aims to explore the specificity of cell types in the aorta of experimental AD using scRNA-seq. We successfully identified 15 clusters of cells which were further divided into nine types of cells based on their marker genes, including Fibroblast, Endothelial, SMC, B cells, Macrophage, DC,T-NK cells, Mast cells, and Neutrophil. We noticed that there were significant changes in SMCs in the formation of AD. Besides, our previous study found that the expression of EMMPRIN in SMCs can promote the formation and progression of AD (Chen et al., 2009). Therefore, we focus on SMCs for further analysis.

Seven core genes were identified in SMCs. ACTA2, is a smooth muscle actin, ranks first among the four algorithms. The mutation of ACTA2 is closely related to aortic dissection (Cooper and Brown, 2017; van de Laar et al., 2019).The second key gene IL6, encodes a cytokine that functions in inflammation and the maturation of B cells, the inflammatory mechanism plays a key role in the development and progression of AD (Lu et al., 2021).CTGF, is a Protein Coding gene, and has important pro-inflammatory and pro-fibrotic characteristic (Morales et al., 2018), which play an important role in the progression of AD (Bode-Jänisch et al., 2012; Bersi et al., 2017; Zhao and Bie, 2020). BGN, encodes a member of the small leucine-rich proteoglycan (SLRP) family of proteins, which plays a role in bone growth, muscle development and regeneration, and collagen fibril assembly in multiple tissues (Fisher et al., 1991; Didangelos et al., 2010). ITGA8 mediate numerous cellular processes including cell adhesion, cytoskeletal rearrangement, and activation of cell signaling pathways (Gaudet et al., 2011), The study of organ injury and fibrosis in rat and mouse models showed that ITGA8 was expressed in vascular SMCs and stromal cells after injury (Nishimura et al., 1991), this suggests that it may play a similar role in the formation of AD. THBS1 is an adhesive glycoprotein that mediates cell-to-cell and cell-to-matrix interactions (Mumby et al., 1984; Staniszewska et al., 2007), In addition, THBS1 can activate TGFβ and matrix metalloproteinase (MMP), which mediates the interaction between cells and matrix and participates in angiogenesis, proliferation and platelet aggregation (Savarimuthu Francis et al., 2011).The finally gene CDH5, encodes a classical cadherin of the cadherin superfamily, it is essential for vascular integrity and endothelial function (Mao et al., 2013). The GO and KEGG enrichment analysis also displayed that the above genes play an crucial role in regulating extracellular matrix and mediating inflammatory signaling pathways.

In single-cell trajectory analysis, we identified 8 subtypes of SMCs. SMC5, SMC6, and SMC7 are the main component of vascular smooth muscle in AD, After GO enrichment analysis of vascular SMCs, we consider that SMC2 is a stromal vascular smooth muscle cell, which is mainly involved in the change of extracellular matrix function. Extracellular matrix remodeling occurs with the increase of aortic stiffness and the gradual weakening of aortic wall, resulting in AD (Bhushan et al., 2019),Therefore, SMC2 is an early cell in the formation of AD.SMC6 is related to the signal transduction of calcium ions, so we consider it as a signal transduction type SMC. In a clinical study, calcium levels in elderly patients with acute AD may help to change normal endothelial physiological function (Vianello et al., 2017), In addition, studies have confirmed human vascular smooth muscle cells (HASMCs) can regulate the functional activity of SMCs through intracellular calciu (Sun et al., 2017). Therefore, SMC6 is considered to play a role in phenotypic transformation in the middle stage of AD formation.SMC7 is related to endoderm formation, cytolastmm and structure morphogenesis, so it may be a transitional SMC before final morphology. SMC5 is associated to the secretion of inflammatory factors, and macrophage-induced inflammation are critical for aortic dissection (Li et al., 2017), exacerbated inflammation contributes to the formation of AD and eventually leads to the tearing of blood vessels (Lian et al., 2019; Wu et al., 2021),Therefore, SMC5 may play a major role in AD rupture.

In summary, our study successfully constructed a mouse model of AD using Ang II, and to our knowledge, this is the first study that using scRNA-seq to reveal the cellular components in mouse AD tissues at a single-cell level. Our research confirmed that SMCs contribute a significant role in the formation of AD, and successfully identified seven key genes that associated with extracellular matrix function in SMCs, namely ACTA2, IL6, CTGF, BGN, ITGA8, THBS1, and CDH5, providing us new therapeutic targets for treatment of AD. We also found the changes of four main cell states of SMCs from matrix type to inflammatory type during the formation of AD, which improved our understanding of the pathogenesis and underlying molecular mechanism in the formation of AD.

## Data Availability

The datasets presented in this study can be found in online repositories. The names of the repository/repositories and accession number(s) can be found below: Gene Expression Omnibus, accession number GSE203594.

## Supplementary Material

Supplementary Figure S1 Smooth muscle cell subtype marker.
