# Supplementary figures and images for: Single-Cell RNA Sequencing Reveals Smooth Muscle Cells Heterogeneity in Experimental Aortic Dissection
Source: Front Genet. 2022 Aug 11;13:836593. doi: 10.3389/fgene.2022.836593 (PMC9403608; doi:10.3389/fgene.2022.836593)

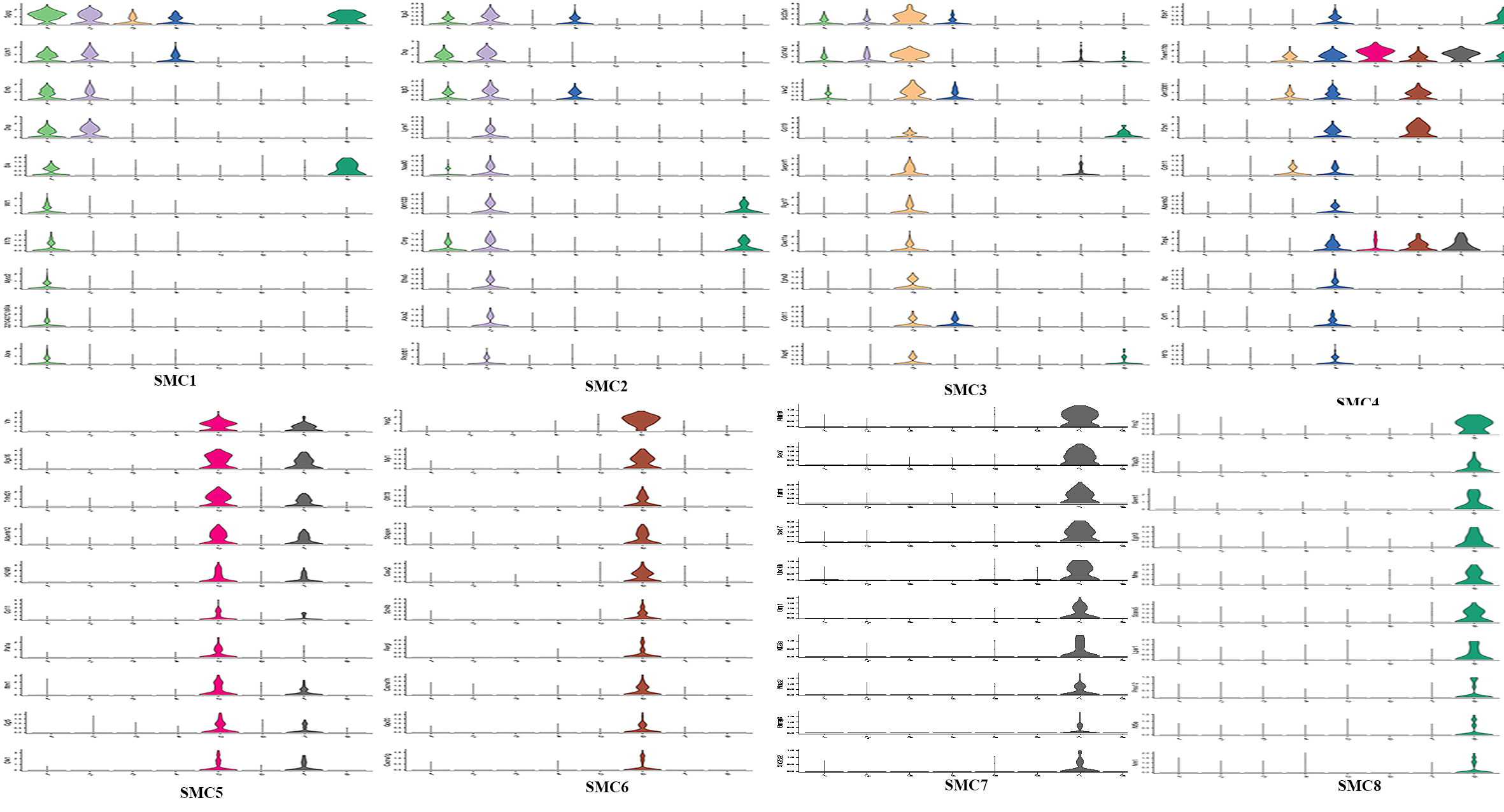

Supplement: Supplementary file 1 [file Image1.tif]
